# Supplementary material for: Epigenetic Induction of Secondary Metabolites Production in Endophytic Fungi Penicillium chrysogenum and GC-MS Analysis of Crude Metabolites with Anti-HIV-1 Activity
Source: Microorganisms. 2023 May 26;11(6):1404. doi: 10.3390/microorganisms11061404 (PMC10305084; doi:10.3390/microorganisms11061404)
Supplement: Supplementary file 1 [file microorganisms-11-01404-s001.zip › microorganisms-2388495-supplementary.pdf]

Epigenetic induction of secondary metabolites production in endophytic fungi Penicillium chrysogenum and GC-MS analysis of crude metabolites with anti-HIV-1

John P. Makhwitine1, Hezekiel M. Kumalo3, Sizwe I. Ndlovu4* Nompumelelo P. Mkhwanazi2*

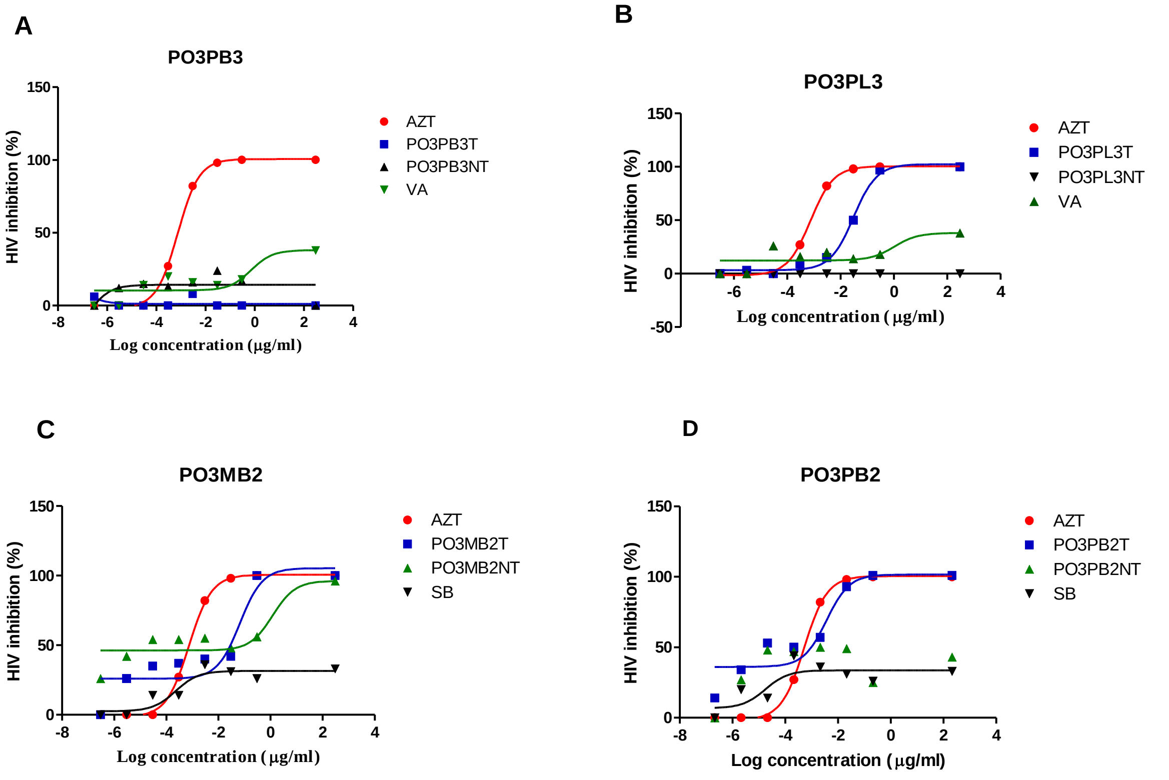

Figure S1: Dose-response curve showing anti-HIV-1 activities of valproic acid treated (PO3PB3x and POPL3) (A and B) and sodium butyrate-treated (PO3MB2T and PO3PB2T) (blue) (C and D) and untreated crude extracts from *P. chrysogenum* (green) tested in TZM-bl cell lines infected with HIV-1 NL4.3. Valproic acid (VA) and Sodium butyrate (SB) extract without fungal culture (black) was included as a negative control, while AZT (red) was used as a positive control. Inhibitory concentration at 50% inhibition (μg/mL). Y axis indicate the percentage HIV-1 inhibition and x axis show the log concentration of the crude extracts. The inhibitory concentration at 50% of the crude extracts were determined. Valproic acid PO3PB3T (no inhibition), PO3PB3NT (IC_{50}= 0.178ug/ml), PO3PL3sT (IC_{50}=0.306 ug/ml). Sodium butyrate PO3MB2T (IC_{50}=0.664ug/ml) and PO3MB2NT (IC_{50}= 1.2ug/ml); PO3PB2T IC_{50}= 0.0361ug/ml) and PO3PB2NT (IC_{50}= not determined).

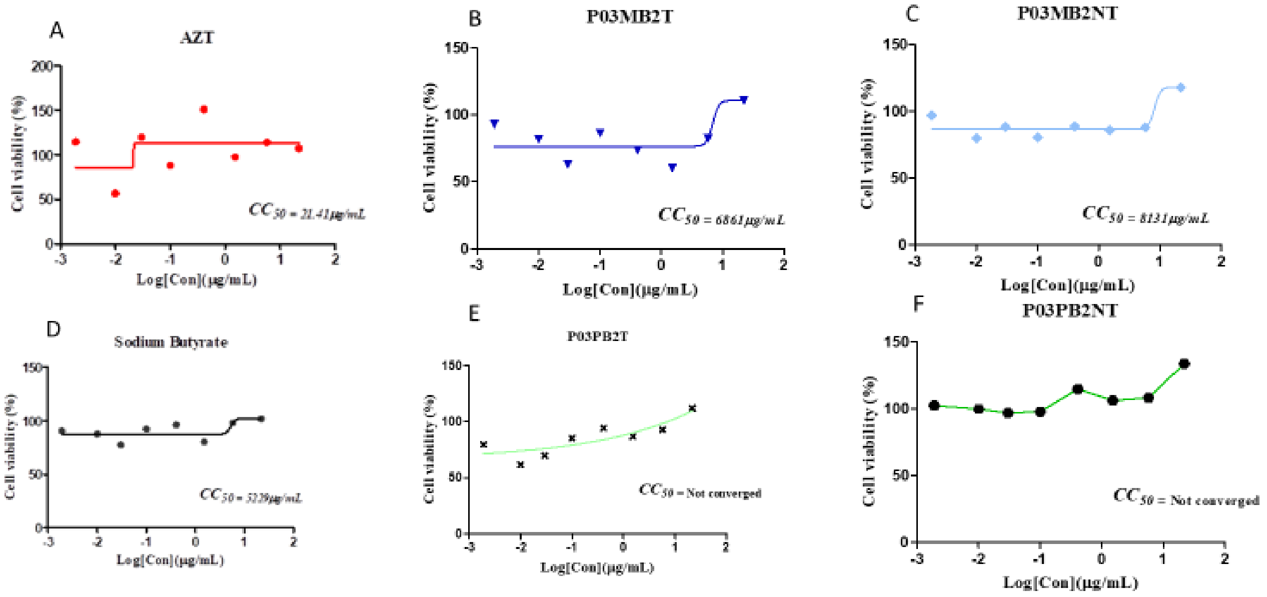

Figure S2: Dose-response curves representing the cytotoxic effects of the crude extracts with their cell cytotoxicity at 50%. Panel A showed the CC_{50} of AZT= 21,41 ug/ml and Panel B and C showed P03MB2T and P03MB2NT CC_{50} respectively. Panel D showed sodium butyrate CC_{50} and Panel E and F showed P03PB2T and P03PB2NT respectively.

Table S1: GC-MS compounds identified from sodium butyrate fractions produced from HLB, MCX, and Max cartridges.

RT (min)	Peak Area (%)	SB Fraction HLB<br>Name of compound	RT (min)	Peak Area (%)	SB Fraction MCX<br>Name of compound	RT (min)	Peak Area (%)	SB Fraction MAX<br>Name of compound
3.550	48.39	1,2-Cyclobutanedicarbonitrile, cis-	3.660	5.72	Propanenitrile	3.515	9.94	1-Buten-3-yne
3.698	3.25	Propanenitrile	4.230	10.76	Cyclotrisiloxane, hexamethyl-	3.545	38.97	1,2-Cyclobutanedicarbonitrile, cis-
4.279	4.17	Cyclotrisiloxane, hexamethyl-	6.293	7.53	Cyclotetrasiloxane, octamethyl-	3.683	1.86	Propanenitrile
6.043	2.35	Cyclotetrasiloxane, octamethyl-	12.111	12.92	Quinoline, 1,2-dihydro-2,2,4-trimethyl-	4.251	8.67	Cyclotrisiloxane, hexamethyl-
6.299	3.69	Cyclotetrasiloxane, octamethyl-	14.790	3.72	Ethanol, 2-(eicosyloxy)-	6.299	2.54	Cyclotetrasiloxane, octamethyl-
11.745	3.76	2,5-Piperazinedione, 3,6-bis(2-methylpropyl)-	15.351	3.16	Indane-4-carbonitrile, 2,2,5,7-tetramethyl-1-oxo-	8.189	0.49	Cyclopentasiloxane, decamethyl-
12.105	5.09	Quinoline, 1,2-dihydro-2,2,4-trimethyl-	15.935	3.44	1,1,3,3-Tetramethyl-1,3-bis[(3Z)-non-3-en-1-yloxy]dis	12.110	3.92	Quinoline, 1,2-dihydro-2,2,4-trimethyl-
12.385	2.89	1-Heptafluorobutyryloxydecane	16.072	3.63	Eicosanoic acid, 2,3-bis[(trimethylsilyl)oxy]propyl ester	12.411	0.94	Trichloroacetic acid, decyl ester
14.796	1.92	Ethanol, 2-(tetradecyloxy)-	16.256	4.83	Pyrrolo[1,2-a]pyrazine-1,4-dione, hexahydro-3-(2-	14.525	0.75	Decane, 4-ethyl-
15.360	1.20	2,8-Dibenzofurandiamine	17.304	5.73	7,9-Di-tert-butyl-1-oxaspiro(4,5)deca-6,9-diene	16.246	2.98	Pyrrolo[1,2-a]pyrazine-1,4-dione, hexahydro-3-(
16.259	2.34	Pyrrolo[1,2-a]pyrazine-1,4-dione, hexahydro-3-(	18.006	16.72	Pyrrolo[1,2-a]pyrazine-1,4-dione, hexahydro-3-(	17.303	1.74	7,9-Di-tert-butyl-1-oxaspiro(4,5)deca-6,9-diene-2,
17.295	2.33	7,9-Di-tert-butyl-1-oxaspiro(4,5)deca-6,9-diene	18.135	12.13	Dibutyl phthalate	17.675	1.45	Pyrrolo[1,2-a]pyrazine-1,4-dione, hexahydro-3-(2-
17.999	7.30	Pyrrolo[1,2-a]pyrazine-1,4-dione, hexahydro-3-(2-	18.895	9.72	Triethylene glycol monododecyl ether	17.998	10.54	Pyrrolo[1,2-a]pyrazine-1,4-dione, hexahydro-3-
18.140	3.46	Dibutyl phthalate				18.135	3.01	1,2-Benzenedicarboxylic acid, bis(2-methylpropyl) ester
18.904	2.96	Diethylene glycol monododecyl ether				18.919	3.49	Diethylene glycol monododecyl ether
23.310	2.47	1H-Imidazole-4,5-dicarboxamide, 1-benzyl-				23.339	8.69	Pyrrolo[1,2-a]pyrazine-1,4-dione, hexahydro-3-
23.384	2.42	N-(2,6-Dichlorophenyl)-3-methylbenzamide						
